# Development of a Broad-Range 23S rDNA Real-Time PCR Assay for the Detection and Quantification of Pathogenic Bacteria in Human Whole Blood and Plasma Specimens

**DOI:** 10.1155/2013/264651

**Published:** 2013-03-17

**Authors:** Paolo Gaibani, Mara Mariconti, Gloria Bua, Sonia Bonora, Davide Sassera, Maria Paola Landini, Patrizia Mulatto, Stefano Novati, Claudio Bandi, Vittorio Sambri

**Affiliations:** ^1^Operative Unit of Clinical Microbiology, St. Orsola-Malpighi University Hospital, 40138 Bologna, Italy; ^2^Department of Haematology and Oncology “L. and A. Seragnoli”, Unit of Clinical Microbiology, Regional Reference Centre for Microbiological Emergencies (CRREM), St. Orsola-Malpighi Hospital, University of Bologna, 9 Via G. Massarenti, 40138 Bologna, Italy; ^3^DIVET, University of Milan, 20100 Milan, Italy; ^4^Fondazione IRCCS Policlinico San Matteo, 27100 Pavia, Italy

## Abstract

Molecular methods are important tools in the diagnosis of bloodstream bacterial infections, in particular in patients treated with antimicrobial therapy, due to their quick turn-around time. Here we describe a new broad-range real-time PCR targeting the 23S rDNA gene and capable to detect as low as 10 plasmid copies per reaction of targeted bacterial 23S rDNA gene. Two commercially available DNA extraction kits were evaluated to assess their efficiency for the extraction of plasma and whole blood samples spiked with different amount of either *Staphylococcus aureus* or *Escherichia coli*, in order to find the optimal extraction method to be used. Manual QIAmp extraction method with enzyme pre-treatment resulted the most sensitive for detection of bacterial load. Sensitivity of this novel assay ranged between 10 and 10^3^ CFU per PCR reaction for *E. coli* and *S. aureus* in human whole blood samples depending on the extraction methods used. Analysis of plasma samples showed a 10- to 100-fold reduction of bacterial 23S rDNA in comparison to the corresponding whole blood specimens, thus indicating that whole blood is the preferential sample type to be used in this real-time PCR protocol. Our results thus show that the 23S rDNA gene represents an optimal target for bacteria quantification in human whole blood.

## 1. Introduction

Blood culture (BC) is the most widely used method for the diagnosis of bloodstream bacterial infections (BSIs) [1]. Major limitations of culture techniques are the intrinsic poor cultivability (or noncultivability) of some bacteria and the inhibitory effects of concurrent antibiotic therapy. In addition, the turn-around time of BC ranges from 24 to 72 hours, which implies that results might become available too late to be of clinical utility [2]. In recent years, molecular methods have been proposed as additional diagnostic tools for BSIs [2, 3]. Several studies reported the development and clinical assessment of broad-range real-time PCR protocols, capable of rapid detection and identification of a vast proportion of cultivable and uncultivable bacteria, from different types of biological samples [4–12]. The majority of the broad-range real-time PCRs use a single pair of universal primers to identify bacteria through the amplification of the 16S ribosomal DNA (16S 0072DNA), given the high level of homology of this gene throughout the bacterial species diversity [12]. The amplification of the 16S rDNA has been described as a specific and sensitive tool to identify and quantify different microorganisms depending on the specific protocol used [7, 13–17]. A major pitfall of 16S-based panbacterial primers is their cross-reactivity with human ribosomal RNA genes; to overcome the problem, Kommedal and coworkers proposed a 16S rDNA-based dual-priming protocol [18]. In addition to 16S rDNA, the gene coding for the large subunit ribosomal RNA (23S rDNA) has also been targeted for the development of PCR methods for bacterial detection, but only a limited number of studies evaluated the utility of 23S-based panbacterial primers [4, 19], and no studies have so far exploited this target for BSI monitoring. The aim of our study was to develop a novel 23S rDNA-targeted real-time pan-bacterial PCR method, suitable for the detection of a wide range of bacterial species, for the monitoring of BSIs. In addition, since the amount of bacterial DNA detected in blood from healthy subjects is reported to be highly variable and profoundly influenced by the use of whole blood or plasma [4, 7, 20], we tested the suitability of different extraction procedures for the isolation of bacterial DNA from blood-derived specimens.

## 2. Materials and Methods

### 2.1. Design of the 23S rDNA Universal Primers

Complete 23S rDNA sequences from 50 bacterial species, spanning the eubacterial diversity, were retrieved from the NCBI database (http://www.ncbi.nlm.nih.gov/). Alignment of the sequences was performed using the MUSCLE software [21] and manually checked. 28S rDNA sequences from *Caenorhabditis elegans*, *Candida albicans*, *Drosophila melanogaster,* and *Homo sapiens* were also included in the alignment, in order to evaluate the specificity for the bacterial rDNA of the designed primers. Primers were manually designed on the obtained alignment, and then evaluated using mfold (http://mfold.rna.albany.edu/?q=mfold) and the Operon oligo analysis tool (http://www.operon.com/tools/oligo-analysis-tool.aspx). The sequences of the 23S rDNA-targeted pan-bacterial primers are PAN23S-F, 5′-TCGCTCAACGGATAAAAG-3′ and PAN23S-R, 5′-GATGAnCCGACATCGAGGTGC-3′; the amplified fragment size is 97 base pairs in *Escherichia coli*. The designed primers were then compared to the nonredundant nucleotide eukaryotic database using the Blast software (http://blast.ncbi.nlm.nih.gov/Blast.cgi), to highlight possible unwanted matches. 

### 2.2. 23S rDNA Real-Time PCR

PCR reactions were effected in a final volume of 25 *μ*L, containing 12.5 *μ*L of SYBR Green PCR Master Mix Reagent (Biorad-Hercules, CA, USA), 250 nM of each primer, and 5 *μ*L of the extracted DNA solution. PCR was performed in an IQ5 thermocycler (Biorad-Hercules, CA, USA) with an initial step of 5 min at 95°C, followed by 40 cycles of 15 s at 95°C and 30 s at 58°C. After PCR amplification, the melting curve was established by increasing the temperature from 55°C to 95°C.

### 2.3. Bacterial Isolates

A panel of 47 different bacterial isolates, 20 Gram-positive, and 27 Gram-negative was included in the study (Table 1). These strains were either obtained from routine cultures, identified at the Unit of Clinical Microbiology, St. Orsola Malpighi Hospital, or obtained from the bacterial collection at the same Institution (BACSO). A cell suspension containing 10^8^ CFU/mL was obtained from each bacterial isolate, and the DNA was extracted using described protocols [22]. PCR products obtained from bacterial cultures were then sequenced to verify whether just bacterial DNA had been amplified and cloned. In addition, five eukaryotic species from the genus *Candida* (*Candida albicans, Candida glabrata, Candida tropicalis, Candida parapsilosis,* and *Candida guilliermondii*) were included in the study. 

### 2.4. PCR Sensitivity Test

An external standard for absolute quantification (i.e., the target 23S rDNA gene fragment, cloned into a plasmid vector) was prepared. PCR was effected on *Staphylococcus aureus* DNA using the above described primers PAN23S-F and PAN23S-R according to standard PCR conditions. The band resolved on a 2% agarose gel was excised and the PCR product was then purified, quantified, and cloned using the pGEM T-easy vector (Qiagen, Basel, Switzerland) according to manufacturers' instructions. Ten randomly selected clones were sequenced with ABI technology. A plasmid containing the 23S rDNA insert was purified from one of the clones, using the QIAprep Spin Miniprep Kit (Qiagen, Basel, Switzerland). After quantification, a serial dilution of the plasmid was used to assess the sensitivity of the above PCR assay, with plasmid at concentrations ranging from 10^8^ to 10^1^ copies per reaction, to generate the standard curve.

### 2.5. DNA Extraction from Whole Blood Spiked with Gram-Positive and Gram-Negative Bacteria

Tenfold serial dilution of bacteria in blood was prepared, by spiking fresh K_3_EDTA blood samples with *S. aureus* ATCC 25923 or *E. coli* ATCC 25922 (as representative strains for Gram-positive and Gram-negative bacteria), to obtain final concentrations of bacteria ranging from 10^7^ CFU per mL to 10 CFU per mL of blood. A written informed consent was obtained from each blood donor following the ethical rules of the St. Orsola Hospital Blood Bank in Bologna. As a control, identical series were prepared in phosphate buffer saline (PBS). Standard 100 *μ*L volumes from each sample of these spiked series (blood or PBS) were subjected to DNA extraction, using the different protocols reported below. Two different commercially available methods were used following the manufacturers' instructions: the automated nucleic acid extractor NucliSens EasyMag (BioMerieux, Marcy l'Etoile, France) and the manual QIAmp DNA blood mini kit (Qiagen, Basel, Switzerland). As a third option, the following modification of the QIAmp DNA blood mini kit was also used: 100 *μ*L of fresh whole blood or PBS spiked series were preincubated with 90 *μ*L of the enzyme solution buffer (20 mg/mL lysozyme, 20 mM Tris HCl, 2 mM EDTA, 1% Triton). After 2 h of incubation at 37°C, the mixture was incubated at 56°C for 2 h with addition of 10 *μ*L of proteinase K, at a concentration of 20 mg/mL (Sigma-Aldrich, St. Louis, MO, USA) and 100 *μ*L of AL Buffer. Then, the mixture (of 300 *μ*L) was subjected to DNA extraction with the QIAmp DNA blood mini kit. The DNA obtained using the three different procedures was eluted to a final volume of 50 *μ*L. In summary, these 50 *μ*L of eluted DNA derived from 1/10 of the blood spiked with the above number of CFUs (i.e., 10^6^–10^0^ CFU in each 50 *μ*L elution). This implies that the five microliters used as template DNA for real-time PCR contained bacterial DNAs derived from 10^5^–10^−1^ CFU. A series of blood samples spiked as above with *E. coli* or *S. aureus* were processed for plasma preparation: the spiked series of blood samples were incubated for 2 hours at room temperature (RT) and then centrifuged at 400 g for 15 minutes at RT. DNA was then extracted as above, using the three different procedures. Each extracted DNA was tested by real-time PCR, and the *CT *values were applied to the standard curve generated in the same experiment to obtain the corresponding copy number of bacterial 23S rDNA gene targets in each reaction. Additionally, real-time PCR was performed on DNA extracted from blood from healthy donors, used as negative control.

## 3. Results

### 3.1. Specificity and Sensitivity of the 23S Real-Time PCR Assay

**Figure 1:**
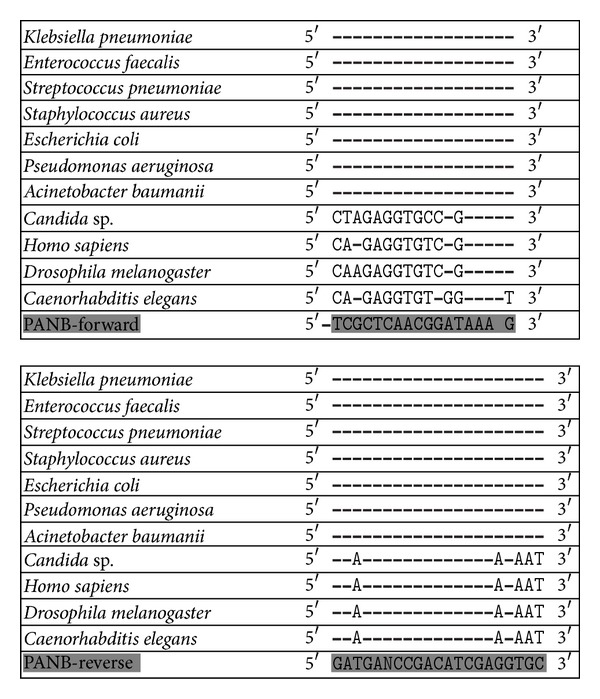
Analysis of PANB-forward and PANB-reverse primers homology sequences against the 23S rDNA of the most common pathogenic bacteria species, *Homo sapiens, Caenorhabditis elegans*, *Candida albicans* and *Drosophila melanogaster* in their binding areas.

The comparison of the designed primers with the sequences from the 50 bacterial species included in the alignment shows an almost complete identity, with no mismatches at the 3′ end, while a high number of mismatches are present in the alignment with the eukaryotic organisms included, that is, *H. sapiens*, *C. elegans*, *C. albicans,* and *D. melanogaster*, as shown in Figure 1.

In order to validate our *in silico* findings, the specificity of the novel 23S rDNA-targeted primers was also evaluated on a total of 20 Gram-positive and 27 Gram-negative bacterial strains (see Table 1 for details) and on five eukaryotic species, from the genus *Candida* (see Section 2). PCR amplification was obtained from the 47 bacterial DNAs, with all the melting curves displaying a sharp peak at the expected Tm; no amplification was obtained from any of the *Candida*-derived DNA (results not shown). Additionally, PCR products obtained from each bacterial amplification were cloned and sequenced as previously described. Sequence of all PCR products obtained was identified as derived from the expected 23S rDNA fragment; these results demonstrate that the developed PCR method specifically detects all the bacterial species tested.

**Figure 2:**
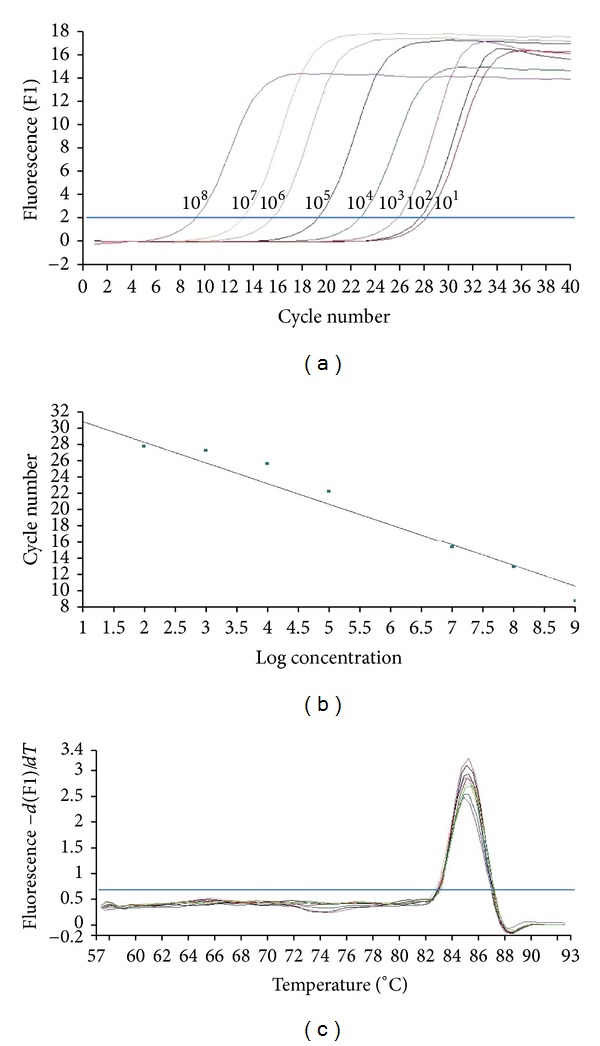
Standard curve amplification of cloned bacterial 23S rDNA plasmid real-time PCR ranging from 10^8^ to 10^1^ copies per reaction. Panel (a) shows the amplification curve constructed by PCR assay. The fluorescence and the corresponding cycle numbers are shown in the vertical and horizontal axes, respectively. Panel (b) shows the relative standard curve ranging from 10^1^ to 10^8^ copies per reaction. Panel (c) shows the corresponding melting curve.

PCR sensitivity was evaluated on a serial dilution of the plasmid containing the 23S rDNA fragment (with each dilution tested in triplicate), at concentrations ranging from 10^8^ to 10^1^ plasmid copies per reaction, to generate a standard curve (*R* value: 0.97; slope value: −2,527; Figure 2). The detection limit of this PCR assay (standard curve method) was 10 copies of 23S rDNA copies/reaction. The melting curve analysis of the 23S rDNA gene product is shown in Figure 2(c). The electrophoresis run for the PCR products showed a unique specific band of 97 pb corresponding to the 23S rDNA gene, thus indicating high specificity. These results demonstrate that the developed PCR method can detect up to 10 copies per reaction, as shown in Figure 2.

### 3.2. DNA Extraction from Whole Blood and PBS Spiked with Gram-Positive and Gram-Negative Bacteria

We evaluated the efficiency of the two commercially DNA extraction methods in PBS and whole blood spiked with *E. coli* and *S. aureus*. Each experiment included a nonspiked whole blood sample as a negative control. In these negative specimens we never observed a completely negative amplification, given the presence of traces of environmental bacterial DNA. Therefore, in order to set up and define the exact number of 23S rDNA copies detected for each sample, the cycle threshold value of the corresponding negative control was used as the edge limit of detection for each run.

**Figure 3:**
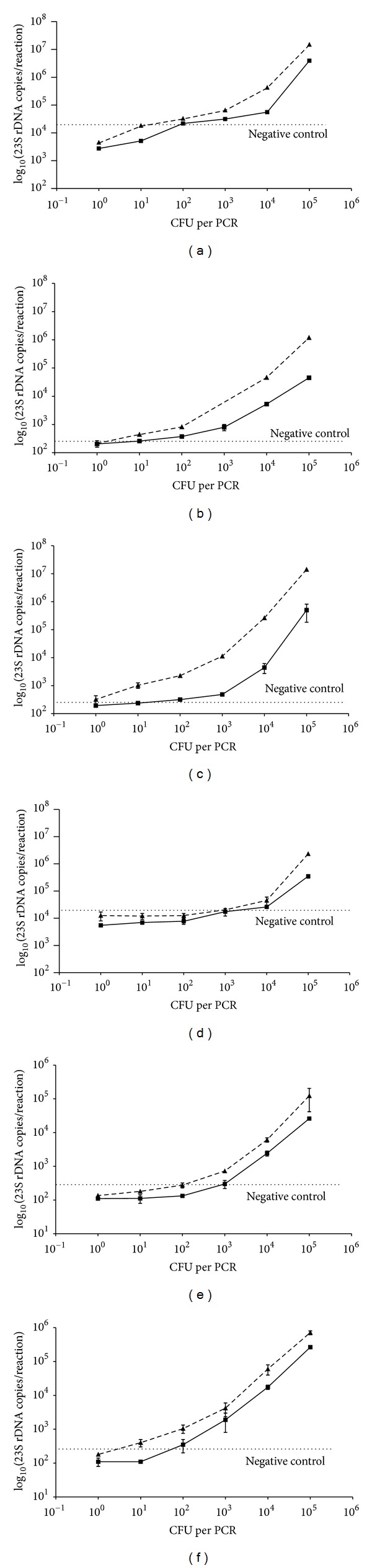
Analytical sensitivities of three different extraction protocols following quantification by the novel 23S rDNA real-time PCR from whole blood (continuous line) and PBS (dotted line) spiked with different Gram-negative (*Escherichia coli*) or Gram-positive (*Staphylococcus aureus*) bacteria ranging from 1 to 10^5^ bacteria per reaction. EasyMag automated extraction protocols with *E. coli* (a) or with *S. aureus* (d) spiked in PBS and in whole blood. QIAmp DNA blood mini kit extraction methods with *E. coli* (b) or with *S. aureus* (e) spiked in PBS and in whole blood; QIAmp DNA blood mini kit extraction with pretreatment with lysozyme and proteinase K with *E. coli* (c) or with *S. aureus* (f), spiked in PBS and in whole blood. Results are representative of three independent experiments, effected on three independent DNA extractions and expressed as mean ± standard deviations.

The examination of sensitivity for detection of *E. coli* in PBS after EasyMag extraction showed a positive signal for concentrations in each PCR reaction in the 10 to 10^2^ CFU range; when blood samples were examined, a 10-fold decrease in the detection sensitivity was observed (Figure 3(a)). When *S. aureus* was tested, the detection sensitivity decreased to 10^3^ CFU per PCR in the case of PBS suspension and to above 10^3^ CFU per PCR when whole blood specimens were evaluated, as shown in Figure 3(d). 

The QIAmp DNA blood mini kit extraction method showed a detection limit for *E. coli* of 1 CFU per PCR in PBS and of 10 CFU per PCR in whole blood samples (Figure 3(b)). The lowest detectable concentrations of *S. aureus* in PBS and whole blood specimens were 10^2^ and 10^3^ CFU per PCR, respectively (Figure 3(e)).

When the pre-treatment step with lysozime and proteinase K was introduced in the QIAmp extraction protocol the PCR sensitivity rose 10-fold for *S. aureus* (Figure 3(f)) whereas no increase was demonstrated for *E. coli* (Figure 3(d)). In particular, the detection limit for *E. coli* was 1 and 10 CFU per PCR in PBS and in whole blood, respectively. The detection limit for *S. aureus* 23S rDNA ranged from 1 to 10 CFU per PCR and 10 to 10^2^ CFU per PCR, respectively, for the bacterial suspension in PBS and whole blood. Similar results were shown for whole blood samples by Zucol and coworkers by using a set of primers and a specific probe targeted on the 16S RNA gene with an extraction protocol based on enzymatic [6]. 

### 3.3. Comparison of the Bacterial 23S rDNA Copy Number in Whole Blood and in Plasma

**Figure 4:**
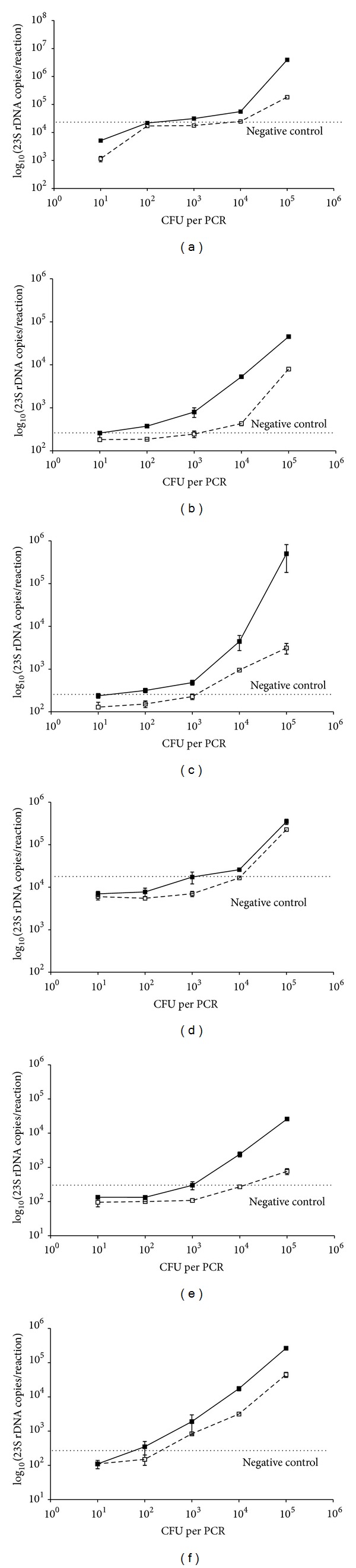
Analytical detection range and sensitivities of different extraction protocols from whole blood (continuous line) spiked with Gram-negative (*E. coli*) or Gram-positive (*S. aureus*) bacteria and their corresponding plasma (dotted line). EasyMag protocols with *E. coli* (a) or with *S. aureus* (d), spiked in plasma and in whole blood. QIAmp DNA blood mini kit extraction with *E. coli* (b) or with *S. aureus* (e), spiked in plasma and in whole blood; QIAmp DNA blood mini kit extraction with pre-treatment with lysozime and proteinase K with *E. coli* (c) or with *S. aureus* (f), in plasma and in whole blood.

Given the potential application of the novel broad-spectrum PCR assay either in plasma or whole blood specimens, we quantified and compared the detection limit of the novel PCR protocol in whole blood specimens spiked with different bacteria and in the derived corresponding plasma samples, as described above. Our results showed that the EasyMag extraction protocol presented a 100-fold reduction of detection sensitivity in the plasma in comparison to the corresponding WB samples (Figures 4(a) and 4(d)). Similarly, the QIAmp DNA blood mini kit with or without the enzymatic pre-treatment showed a similar behavior (Figures 4(b), 4(c), 4(e), and 4(f)). In particular, the QIAmp DNA blood mini kit with pre-treatment showed a positive signal for *E. coli* for as low as 10^1^ and 10^3^ CFU per PCR from whole blood and plasma, respectively (Figure 4(c)), and the minimum detection limit for *S. aureus *ranged from 10^1^ to 10^2^ CFU per PCR and 10^2^ to 10^3^CFU per PCR from whole blood and plasma (Figure 4(f)). Our results showed that the PCR sensitivity was lower in plasma samples than in the corresponding whole blood samples for both *S. aureus* and *E*. *coli*, independently from the extraction method used.

## 4. Discussion

In this study, we developed and assessed the analytical performance of a novel real-time PCR method targeted on a conserved region of the 23S ribosomal DNA gene, for the detection and quantification of a wide range of human pathogenic bacteria in human blood and plasma samples. PCR assays capable of detecting a wide range of human pathogens are nowadays important tools in microbiology laboratories, useful for the detection and identification of infecting bacteria, in particular in the case of blood stream infections. This approach demonstrated its utility in cases of patients receiving ongoing antimicrobial therapy or infected by fastidious/not cultivable bacteria [1, 9]. So far, 16S rDNA has been the most widely used target for these applications. As detailed in the introduction, one concern that was raised against 16S rDNA-based approaches was its possible lack of specificity, for example, the cross-reactivity with human DNA, but this issue was not largely investigated [18, 23–25]. As target for a pan-bacterial PCR, the 23S rDNA region offers some advantages compared to 16S rDNA: (i) a higher content of variable sequence stretches, (ii) the presence of unique insertions/deletions, and (iii) possibility of a better phylogenetic resolution because of a higher sequence variation [19, 26, 27]. 

The novel 23S rDNA-targeted PCR assay described here was able to specifically detect all of the 47 bacterial isolates included in the study that represent an estimated 90% of the reported causes of blood stream infections [6]; no false positive reactions were observed when eukaryotic DNAs of diverse origin were tested. A previous study conducted by Zucol and co-workers showed a sensitivity lower than that reported here, when combined nucleic acids extraction methods were applied to water bacterial suspensions and then followed by a 16SrDNA-targeted broad-spectrum real-time PCR assay [6]. As expected, the performance of that method was lower when applied to detect Gram-positive in respect to Gram-negative bacteria, likely due to the thicker cell wall of the former [6].

The minimal detection limits determined for the novel real-time PCR described here were in the range of 1 to 10^3^ CFU per reaction in whole blood spiked with *E. coli* or *S. aureus*, depending on DNA extraction methods; as expected, this indicates that the DNA purification step can have a profound effect on the final results. The modified QIAmp DNA blood mini kit, with pre-treatment of the samples with proteinase K and lysozyme that contribute to the disruption of the bacterial cell envelopes, led to a sensitivity of the overall procedure that appears superior compared to methods without preenzymatic treatments (see Figure 3).

Some previously published studies showed controversial results for the detection of the bacterial 16S rDNA in blood. In particular, differences were found when whole blood or plasma specimens were evaluated [7, 15, 20, 28]. The effective difference in the amount of bacterial rDNAs detectable in whole blood or plasma samples remains up to today not fully investigated, so that the discussion about the optimal type of samples to be used for the detection of bacterial rDNAs from blood stream specimens is still ongoing [2]. In this study, we tested whole blood samples spiked with either *E. coli* or *S. aureus* and the derived plasma specimens, demonstrating that the detection of 23S rDNA was more effective when whole blood was used.

These results suggest that for an appropriate and effective detection of bacterial 23S rDNA the most promising type of sample is whole blood, while plasma must be considered a second choice, given the lower detection limit. A major weakness of the novel test described in this paper is the short sequence, 97 base pair, used as target for the assay, that makes quite unlikely the possibility to use the amplicons for a successive species identification, based on sequence analysis. On the other hand, it should be considered that in most cases of invasive bacterial disease and in particular in blood stream infections, the bacterial load in biological samples, such as cerebrospinal fluid and blood, is usually quite low and the use of a very sensitive diagnostic method, like the novel PCR assay described in this paper, is highly desirable in order to prove the microbial etiology. A similar approach was recently described by Banada and co-workers, who showed that an increase in the sensitivity of the PCR protocol can be obtained by a decrease in amplicon size [28]. Additional methods, capable of identifying the infective germs could be applied later on in the diagnostic workflow, eventually with additional steps capable of increasing the bacterial load, such as enrichment broth culture or testing on specimens obtained after withdrawal or suspension of the antimicrobial therapy. The presence of bacterial products, including nucleic acids, in the blood is nowadays a well-recognized phenomenon that frequently occurs when pathological conditions allow translocation from highly colonized sites, such as the bowel [29, 30]. One major application of the method in this study could be the quantification of total bacterial DNA in the bloodstream in all the cases in which a sensitive detection of DNA is desired without the need for a precise bacterial species identification.

## Figures and Tables

**Table 1:** Bacterial strains utilized in this study. The strains were either obtained from the bacterial collection of the St. Orsola Hospital (BACSO) or derived from routine workflow. In this last case the procedure for identification are reported in the Qualty Assurance files of the Laboratory.

Microorganisms species	Origin of the isolate
*Acinetobacter baumannii *	Urine
*Acinetobacter lwoffii *	Urine
*Alcaligenes xylosoxidans *	Urine
*Bacteroides fragilis *	Cerebrospinal fluid
*Campylobacter jejuni *	Feces
*Citrobacter braakii *	Abdominal drainage
*Citrobacter freundii *	Blood
*Citrobacter koseri *	Urine
*Corynebacterium jeikeium *	Blood
*Corynebacterium minutissimum *	Blood
*Corynebacterium striatum *	Blood
*Corynebacterium urealyticum *	Blood
*Enterobacter cloacae *	Urine
*Enterobacter aerogene *	Urine
*Enterococcus casseliflavus *	Blood
*Enterococcus faecalis *	Bacso/atcc 29212
*Enterococcus faecium *	Blood
*Enterococcus gallina rum *	Blood
*Escherichia coli *	Bacso/atcc 25922
*Haemophilus influenzae *	Bacso/atcc 49247
*Haemophilus influenzae *	Bacso/atcc 49766
*Hafnia alvei *	Bile
*Klebsiella oxytoca *	Blood
*Klebsiella pneumoniae *	Urine
*Morganella morganii *	Urine
*Nocardia *sp.	Bronchial aspirate
*Proteus mirabilis *	Urine
*Proteus vulgaris *	Bronchial aspirate
*Providencia stuartii *	Urine
*Pseudomonas aeruginosa *	Bacso/atcc 27853
*Pseudomonas luteola *	Bronchial aspirate
*Salmonella *sp. Group B	Feces
*Salmonella *sp. Group C	Feces
*Salmonella *sp. Group D	Feces
*Serratia marcescens *	Urine
*Staphylococcus aureus *	Bacsoatcc 29213
*Staphylococcus epidermidis *	Blood
*Staphylococcus haemolyticus *	Blood
*Staphylococcus hominis *	Blood
*Staphylococcus warneri *	Blood
*Stenotrophomonas maltophilia *	Faringeal swab
*Streptococcus agalactiae *	Uretral swab
*Streptococcus anginosus *	Blood
*Streptococcus mitis *	Blood
*Streptococcus parasanguinis *	Blood
*Streptococcus pyogene *	Faringeal swab
*Streptococcus pneumoniae *	Bacso/atcc 49619

## References

[1] Mylotte JM, Tayara A (2000). Blood cultures: clinical aspects and controversies. *European Journal of Clinical Microbiology and Infectious Diseases*.

[2] Paolucci M, Landini MP, Sambri V (2010). Conventional and molecular techniques for the early diagnosis of bacteraemia. *International Journal of Antimicrobial Agents*.

[3] Gaibani P, Rossini G, Ambretti S (2009). Blood culture systems: rapid detection: how and why?. *International journal of antimicrobial agents*.

[4] Anthony RM, Brown TJ, French GL (2000). Rapid diagnosis of bacteremia by universal amplification of 23S ribosomal DNA followed by hybridization to an oligonucleotide array. *Journal of Clinical Microbiology*.

[5] Rosey AL, Abachin E, Quesnes G (2007). Development of a broad-range 16S rDNA real-time PCR for the diagnosis of septic arthritis in children. *Journal of Microbiological Methods*.

[6] Zucol F, Ammann RA, Berger C (2006). Real-time quantitative broad-range PCR assay for detection of the 16S rRNA gene followed by sequencing for species identification. *Journal of Clinical Microbiology*.

[7] Jiang W, Lederman MM, Hunt P (2009). Plasma levels of bacterial DNA correlate with immune activation and the magnitude of immune restoration in persons with antiretroviral-treated HIV infection. *Journal of Infectious Diseases*.

[8] Matsuda K, Tsuji H, Asahara T, Kado Y, Nomoto K (2007). sensitive quantitative detection of commensal bacteria by rRNA-targeted reverse transcription-PCR. *Applied and Environmental Microbiology*.

[9] Rampini SK, Bloemberg GV, Keller PM (2011). Broad-range 16S rRNA gene polymerase chain reaction for diagnosis of culture-negative bacterial infections. *Clinical Infectious Diseases*.

[10] Yang S, Lin S, Kelen GD (2002). Quantitative multiprobe PCR assay for simultaneous detection and identification to species level of bacterial pathogens. *Journal of Clinical Microbiology*.

[11] Zapater P, Francés R, González-Navajas JM (2008). Serum and ascitic fluid bacterial DNA: a new independent prognostic factor in noninfected patients with cirrhosis. *Hepatology*.

[12] Clarridge JE (2004). Impact of 16S rRNA gene sequence analysis for identification of bacteria on clinical microbiology and infectious diseases. *Clinical Microbiology Reviews*.

[13] Bacchetti De Gregoris T, Aldred N, Clare AS, Burgess JG (2011). Improvement of phylum- and class-specific primers for real-time PCR quantification of bacterial taxa. *Journal of Microbiological Methods*.

[14] Cherkaoui A, Emonet S, Ceroni D (2009). Development and validation of a modified broad-range 16S rDNA PCR for diagnostic purposes in clinical microbiology. *Journal of Microbiological Methods*.

[15] Ferri E, Novati S, Casiraghi M (2010). Plasma levels of bacterial DNA in HIV infection: the limits of quantitative polymerase chain reaction. *Journal of Infectious Diseases*.

[16] Gentili V, Balboni PG, Menegatti E (2011). Panbacterial real-time PCR to evaluate bacterial burden in chronic wounds treated with Cutimed Sorbact. *European Journal of Clinical Microbiology and Infectious Diseases*.

[17] Zemanick ET, Wagner BD, Sagel SD, Stevens MJ, Accurso FJ, Kirk Harris J (2010). Reliability of quantitative real-time PCR for bacterial detection in cystic fibrosis airway specimens. *PLoS ONE*.

[18] Kommedal Ø, Simmon K, Karaca D, Langeland N, Wikera HG (2012). Dual priming oligonucleotides for broad-range amplification of the bacterial 16S rRNA gene directly from human clinical specimens. *Journal of Clinical Microbiology*.

[19] Hunt DE, Klepac-Ceraj V, Acinas SG, Gautier C, Bertilsson S, Polz MF (2006). Evaluation of 23S rRNA PCR primers for use in phylogenetic studies of bacterial diversity. *Applied and Environmental Microbiology*.

[20] Nikkari S, McLaughlin IJ, Bi W, Dodge DE, Relman DA (2001). Does blood of healthy subjects contain bacterial ribosomal DNA?. *Journal of Clinical Microbiology*.

[21] Edgar RC (2004). MUSCLE: multiple sequence alignment with high accuracy and high throughput. *Nucleic Acids Research*.

[22] Epis S, Gaibani P, Ulissi U, Chouaia B, Ricci I (2012). Do mosquito-associated bacteria of the genus Asaia circulate in humans?. *European Journal of Clinical Microbiology & Infectious Diseases*.

[23] Harris KA, Hartley JC (2003). Development of broad-range 16S rDNA PCR for use in the routine diagnostic clinical microbiology service. *Journal of Medical Microbiology*.

[24] Rantakokko-Jalava K, Nikkari S, Jalava J (2000). Direct amplification of rRNA genes in diagnosis of bacterial infections. *Journal of Clinical Microbiology*.

[25] Vandercam B, Jeumont S, Cornu O (2008). Amplification-based DNA analysis in the diagnosis of prosthetic joint infection. *Journal of Molecular Diagnostics*.

[26] Ludwig W, Schleifer KH (1994). Bacterial phylogeny based on 16S and 23S rRNA sequence analysis. *FEMS Microbiology Reviews*.

[27] Pei A, Nossa CW, Chokshi P (2009). Diversity of 23S rRNA genes within individual prokaryotic genomes. *PloS One*.

[28] Banada PP, Chakravorty S, Shah D, Burday M, Mazzella FM, Alland D (2012). Highly sensitive detection of *staphylococcus aureus* directly from patient blood. *PLoS ONE*.

[29] Gómez-Hurtado I, Santacruz A, Peiró G (2011). Gut microbiota dysbiosis is associated with inflammation and bacterial translocation in mice with CCl4-Induced fibrosis. *PLoS ONE*.

[30] Kramski M, Gaeguta AJ, Rajasuriar R (2011). Novel sensitive real-time PCR for quantification of bacterial 16S rRNA genes in plasma of HIV-infected patients as a marker for microbial translocation. *Journal of Clinical Microbiology*.

